# Changes in cecal microbiota community of suckling piglets infected with porcine epidemic diarrhea virus

**DOI:** 10.1371/journal.pone.0219868

**Published:** 2019-07-16

**Authors:** Zhen Tan, Wanting Dong, Yaqun Ding, Xiangdong Ding, Qin Zhang, Li Jiang

**Affiliations:** 1 Key Laboratory of Animal Genetics, Breeding and Reproduction, Ministry of Agriculture & National Engineering Laboratory for Animal Breeding, College of Animal Science and Technology, China Agricultural University, Beijing, P.R. China; 2 College of Animal Science and Technology, Institute of Tropical Agriculture and Forestry, Hainan University, Haikou, P.R. China; University of Illinois at Chicago, UNITED STATES

## Abstract

Diarrhea, caused by porcine epidemic diarrhea virus (PEDV), is a catastrophic gastrointestinal disease among suckling piglets, with high infectivity, morbidity, and mortality, causing huge economic losses to the pig industry. In the present study, we investigated the different microbiota from the cecal mucosa and cecal contents between healthy and PEDV-infected piglets. High-throughput 16S rRNA gene sequencing was performed to explore differences. The results revealed that microbial dysbiosis by PEDV infection occurred in the cecal mucosa and contents of suckling piglets at each microbial taxonomic level. The abundance of pathogenic bacteria associated with diseases, including diarrhea, was increased. The abundance of Fusobacterium was 26.71% and 33.91% in cecal mucosa and contents of PEDV-infected group, respectively, whereas that in the healthy groups was 17.85% and 9.88%. The proportion of Proteobacteria in the infected groups was relatively high (24.67% and 22.79%, respectively), whereas that in the healthy group was 13.13% and 11.34% in the cecal mucosa and contents, respectively. Additionally, the proportion of Bacteroidetes in the healthy group (29.89%, 37.32%) was approximately twice that of the PEDV-infected group (15.50%, 15.39%). “Nitrate reduction”, “Human pathogens diarrhea”, “Human pathogens gastroenteritis”, “Nitrite respiration”, and “Nitrite ammonification” were the enriched functional annotation terms in the PEDV-infected groups. Porcine epidemic diarrhea virus infection increased the proportion of harmful bacteria and decreased the proportion of beneficial bacteria in the cecal mucosa and contents of suckling piglets. Our findings suggest that determining the intestinal microbiota might provide a promising method to prevent PEDV and open a new avenue for future research.

## Introduction

Porcine epidemic diarrhea virus (PEDV), a member of the family Coronaviridae, can cause enteric diseases in swine, infect porcine enterocytes [1], induce acute mucosal damage, blunt intestinal villi, and reduce the thickness of the intestinal walls [2]. The clinical characters of PED include severe enteritis, vomiting, acute diarrhea, anorexia, dehydration, and death; these can occur [2] in pigs of all ages. However, PED is the most serious threat to piglets at the age of one week, with the morbidity and mortality rates between 80% and 100% [3, 4]. During recent years, PED has widely spread globally, particularly in Europe, Asia, and America. A large-scale outbreak of PED in October 2010 in Southern China caused more than one million mortalities of newborn piglets [5]. Further, PEDV has caused serious loss to pig farming industry [3, 4, 6, 7].

In the past, most studies on PED have mainly focused on the molecular characterization of PEDV [8, 9], sequence-based phylogeny analysis, pathogenicity, host immune response to the virus [10, 11], and/or vaccinations, whereas studies on the relationship between gut microbiota are limited. Studies have indicated that the intestinal microflora play physiological, nutritional, and immunological roles in maintaining the gut health of the host [12]; maintain normal function of the intestinal villi [13]; regulate the immune responses [14]; and protect the host from both pathogenic and commensal bacteria [15, 16]. Commensal microbiota can prevent pathogenic invasion by competing for receptors and enteric nutrients [11], stimulating the innate immune system to inhibit pathogens, producing antimicrobial compounds such as bacteriocins [16], and creating a microenvironment that is adverse to enteric pathogens [11, 17, 18]. Increased knowledge on the community structure and functional capacity of the gut microbiota can help discover relationships between microbial functions and the host physiology and metabolism.

In pigs, a few studies have focused on the close relationship between PEDV and intestinal microorganisms. It has been reported that PEDV infection causes the gut microbial dysbiosis, decreasing the proportion of probiotic bacteria and increasing the proportion of pathogenic bacteria. The proportion of members of the phyla Fusobacteria and Verrucomicrobia, the genus *Enterococcus*, and most of the commensal bacteria was higher in PEDV-infected pigs than in healthy ones [19–22]. However, to the best of our knowledge, studies on the microbial structure and function of cecal intestinal contents and cecal mucosa of pigs have not been conducted. In the present study, we investigated the different microbiota from the cecal mucosa and cecal contents between healthy and PEDV-infected piglets. Our results will enhance the understanding of gut microbiota associated with PEDV.

## Materials and methods

### Animal experiments

All the piglets in this study were from a commercial breeding pig farm in Shandong (Binzhou, China). The F1 offspring of Landrace and Yorkshire pigs were used, and they were fed corn-soybean commodity diet under the same conditions. The piglets were selected from five sow stalls of the same farrowing house with symptoms of diarrhea from days 7–9 after the piglets were born.

A few piglets in these stalls exhibited diarrhea symptoms, while others displayed no symptoms. The piglets were then divided into diarrhea and healthy groups. The diarrhea group consisted of piglets displaying symptoms of diarrhea, whereas piglets of the healthy group exhibited no such symptoms. Piglets of both the groups were anaesthetized with excessive sodium pentobarbital and then sacrificed by dissecting the jugular vein. To further confirm the status of PEDV infection in each individual piglet, we used quantitative polymerase chain reaction (qPCR) to detect PEDV in the jejunal mucosa of all the piglets, and thus, confirmed the phenotype of all the individuals. The digesta samples and mucosa tissues from the gut were collected under aseptic conditions within 30 min of euthanasia. The samples were snap freezed in liquid nitrogen. All the samples were collected in sterile tubes and stored in liquid nitrogen until analysis.

The jejunal mucosa samples from all individuals were used to extract virus RNA using the QIAamp viral RNA MINI kit (QIAGEN, Valencia, CA). A pair of specific primers was used for the SYBR Green I qPCR to identify the piglets infected with PEDV [23]. The qPCR was performed in the Roche LightCycler 480 instrument following the manufacturer’s guidelines and cycling conditions using the LightCycler 480 SYBR Green I Master. The reactions were carried out in a total reaction volume of 20 μL containing 1 μL of template, 10 μL of Blue-SYBR-Green mix, 1 μL each of the forward and reverse primers (10 pM/μL), and 7 μL of ddH_2_O (Roche Applied Science, USA). The qPCR for each sample was performed in triplicate. In addition, based on the test results of qPCR and the phenotype of all piglets in both the groups, the samples with a CT value of 0 detected by qPCR were calculated as 40. Thirty samples were collected from the cecal mucosa of the healthy piglets (Ce.MH, n = 5), cecal mucosa of the infected piglets (Ce.MD, n = 10) (S1 Fig), cecal contents of the healthy piglets (Ce.CH, n = 6), and cecal contents of the infected piglets (Ce.CD, n = 9) (S2 Fig).

The microbial genomic DNA was extracted from the samples and purified using the QIAamp DNA Stool Mini Kit (Qiagen Ltd., Germany) following the manufacturer’s instructions. Adequate quantity of high-quality genomic DNA was extracted, and the concentration of DNA was measured using a UV–Vis spectrophotometer (NanoDrop 2000c, USA).

### 16S rRNA gene sequencing and data analysis

The V4 region of the 16S rRNA gene was amplified (515F–806R) by the PCR with the universal bacterial 16S rRNA gene PCR amplicon primers [24]. The PCR was carried out using 30 μL of reaction mixture containing 15 μL of Phusion High-Fidelity PCR Master Mix (New England Biolabs). Mixed PCR products were purified using the GeneJET Gel Extraction Kit (Thermo Scientific) following the manufacturer’s instructions. Sequencing libraries were generated using the NEB Next Ultra DNA Library Prep Kit for Illumina (NEB, USA) following the manufacturer’s recommendations. The library was sequenced on an Illumina HiSeq2500 platform and 250-bp paired-end reads were generated.

Effective tags from the original DNA fragments were merged using FLASH (V1.2.7, http://ccb.jhu.edu/software/FLASH/) [25]. Quality of the raw tags was controlled using QIIME (V1.9.1, http://qiime.org/scripts/split_libraries_fastq.html) [26]. Chimeric sequences were removed using UCHIME (http://www.drive5.com/usearch/manual/uchime_algo.html) [27]. Sequence clustering was performed using UPARSE (V7.0.1001, http://drive5.com/uparse/) [28]. Sequences with ≥ 97% similarity were assigned to the same operational taxonomic units (OTUs). The OTUs were annotated with taxonomic information compared with the SSU rRNA database [29] from SILVA (http://www.arb-silva.de/) [30]. The effective tags of each sample were calculated, and the sample with the least amount of effective tags was used as the standard for homogenization. Subsequent alpha and beta diversity analyses were all based on the data obtained after homogenization.

Alpha and beta diversity analyses were performed using QIIME (V1.9.1) [26]. Heatmaps were plotted to cluster samples from the different groups. Phylum and family relative abundance were represented by stacked bar charts. Weighted UniFrac distances were used for principal coordinate analysis (PCoA) [31]. Pictures were drawn and statistical analyses were carried out using R (http://www.R-project.org). The linear discriminant analysis effect size (LEfSe) was used for the quantitative analysis of biomarkers within different groups [32]. Microbial functional annotation was predicted using FAPROTAX [33]. The data were deposited in the National Center for Biotechnology Information’s Short Read Archive with the accession no. SRP162202.

### Ethics approval

All animal experiments were reviewed and approved by the Institutional Animal Care and Use Committee of China Agricultural University and performed in accordance with the Guidelines for Experimental Animals of the Ministry of Science and Technology (Beijing, China). All the methods were in accordance with the guidelines approved by the Quality Supervision, Inspection, and Quarantine of the People’s Republic of China (GB/T 17236–2008). The Animal Welfare Committee of China Agricultural University approved all experimental protocols (permit number: DK996).

## Results

### Microbiota profile of cecal mucosa and contents

**Fig 1:**
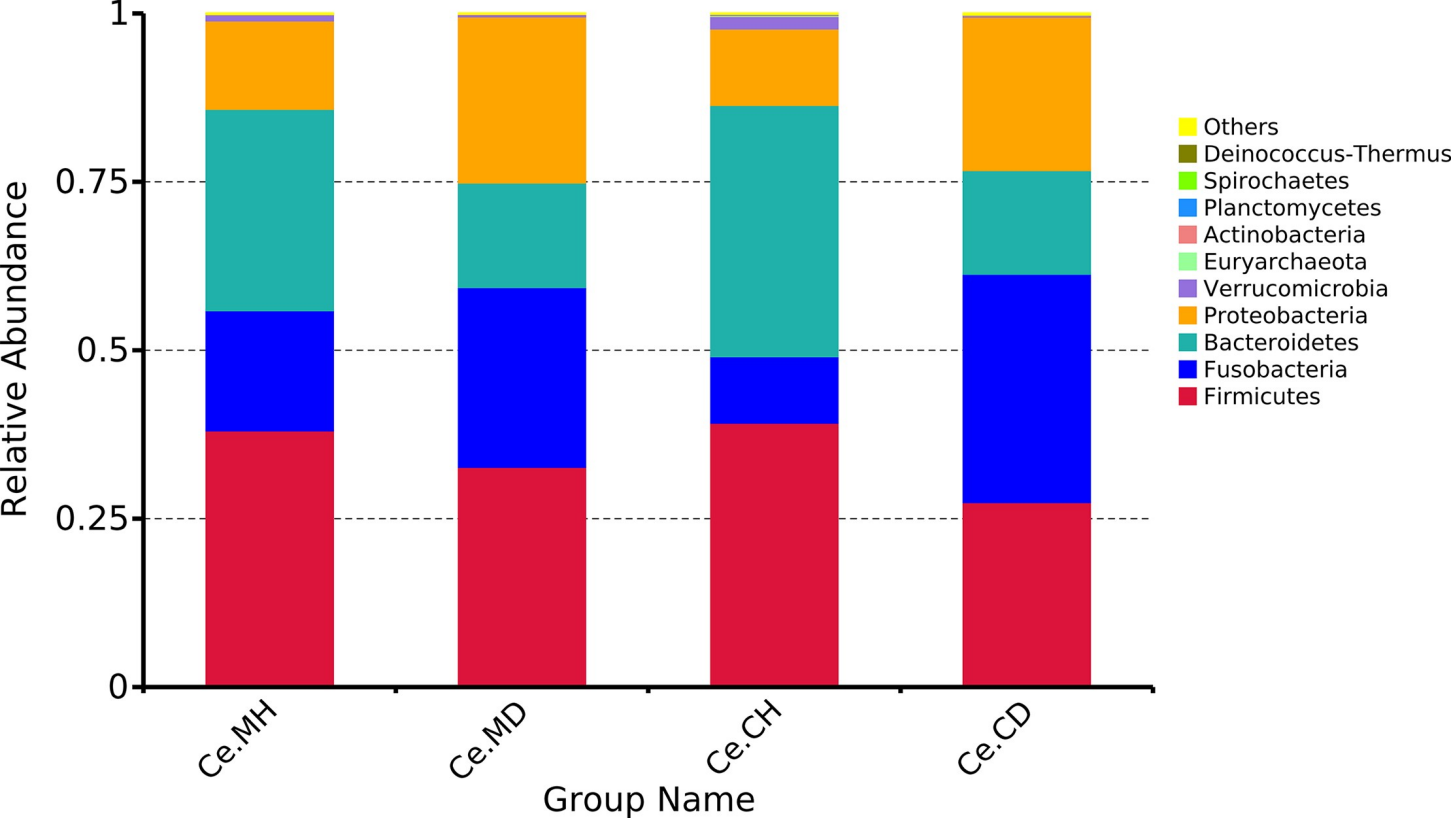
Average relative abundance of cecal microbial species at the phylum level. Ce.CD, cecal content of infected suckling piglets, Ce.CH, cecal content of infected healthy piglets, Ce.MD, cecal mucosa of infected suckling piglets, Ce.MH, cecal mucosa of healthy suckling piglets.

An average of 115,546 sequences per sample (range: 105,419–126,244) of 16S rRNA V4 region was generated. The average length of effective tags was 253 bp. The taxonomic analysis indicated that the first four phyla accounted for more than 95% of the bacteria in each group-the Firmicutes, Bacteroidetes, Proteobacteria, and Fusobacteria (Fig 1). At the level of dominant phylum, the difference in microorganism structure between the healthy and infected groups was more obvious than that between the cecal content and mucosa. Among them, the proportion of Firmicutes in the cecal mucosa and contents in the healthy groups was 38.11% (Ce.MH) and 39.24% (Ce.CH), whereas that in the infected groups was 32.68% (Ce.MD) and 27.45% (Ce.CD), respectively. In the cecal mucosa and contents, the proportion of Bacteroidetes in the healthy groups (29.89% and 37.32%) was approximately twice that of the infected groups (15.50% and 15.39%), respectively. On the contrary, the proportion of Proteobacteria in the infected groups was relatively high, with 24.67% and 22.79% in the cecal mucosa and contents, whereas that in the healthy groups was 13.13% and 11.34%, respectively. Similarly, the percent of Fusobacteria in the cecum mucosa and contents of infected groups was 26.71% and 33.91%, whereas that in the healthy groups was 17.85% and 9.88%, respectively.

**Fig 2:**
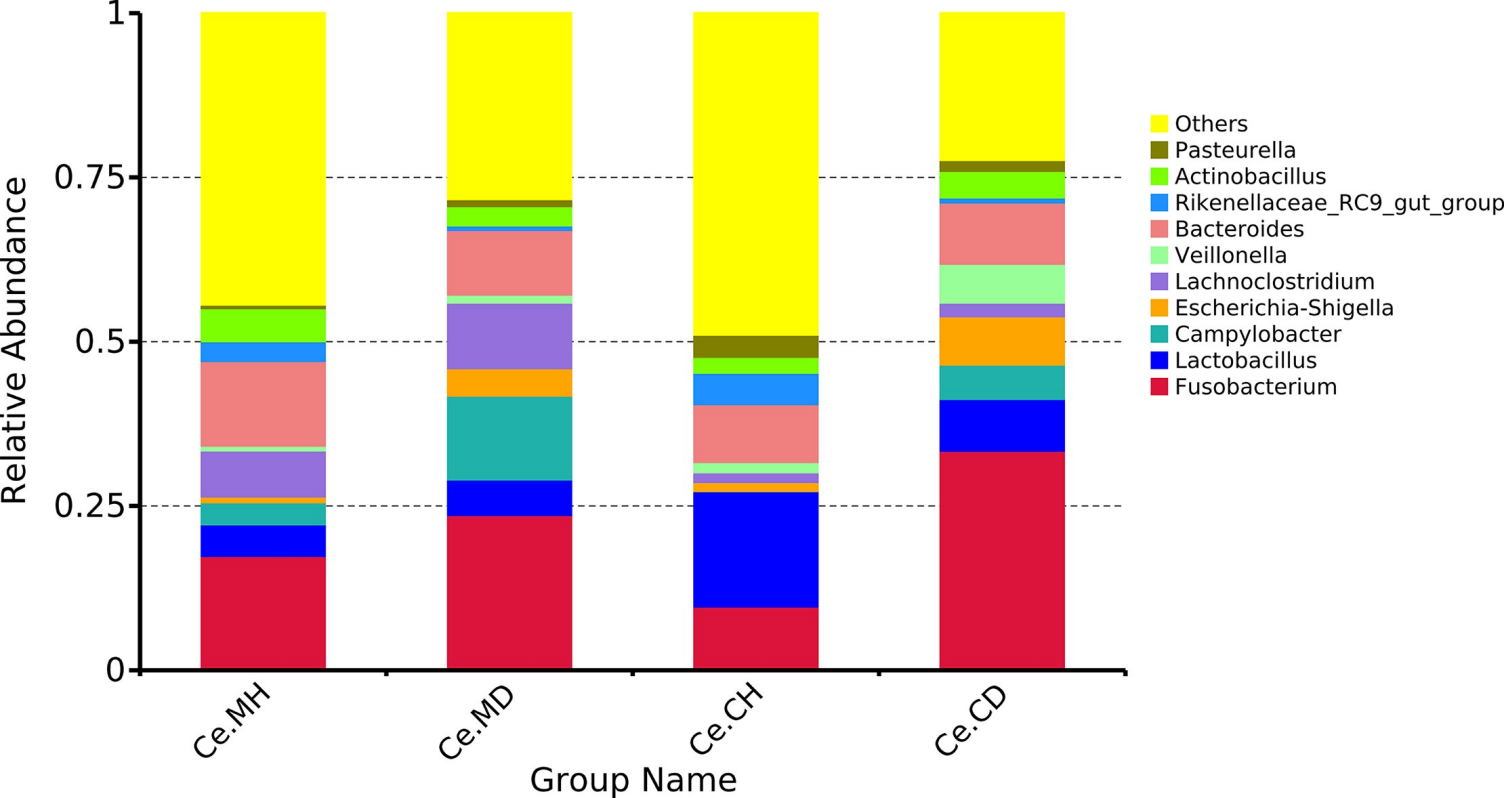
Average relative abundance of cecal microbial species at the genus level. Ce.CD, cecal content of infected suckling piglets, Ce.CH, cecal content of infected healthy piglets, Ce.MD, cecal mucosa of infected suckling piglets, Ce.MH, cecal mucosa of healthy suckling piglets.

To further investigate the microbiota distribution in the healthy and infected groups, we performed genera level analyses. The top ten genera among four groups including *Fusobacterium*, *Bacteroides*, *Lactobacillus*, *Campylobacter*, *Lachnoclostridium*, and *Escheria–Shigella* are shown in Fig 2. The proportion of *Bacteroides* in the four groups was approximately 10%, and there were no significant difference among them. The proportion of *Fusobacterium* in the infected groups was 23.62% (Ce.MD) and 33.39% (Ce.CD), whereas that in the healthy groups was 17.39% (Ce.MH) and 9.68% (Ce.CH). The proportion of *Lactobacillus* in the Ce.CH group was considerably higher than that in the other three groups, reaching 17.57%, whereas the proportion of *Campylobacter* in the infected groups was significantly higher than that in the healthy groups, especially in the Ce.MD group, reaching 12.74%. In the cecal mucosa and contents, the proportion of microorganisms was also different. The proportion of *Lactobacillus* in the cecal contents was relatively higher than that in the mucosa, whereas the proportion of *Lachnoclostridium* in the mucosa was significantly higher than that in the cecal contents. “Others” included the unidentified genera, and their relative proportion was less than that of the first 10 genera.

### Differences in the cecal microbiota between the healthy and infected groups

**Fig 3:**
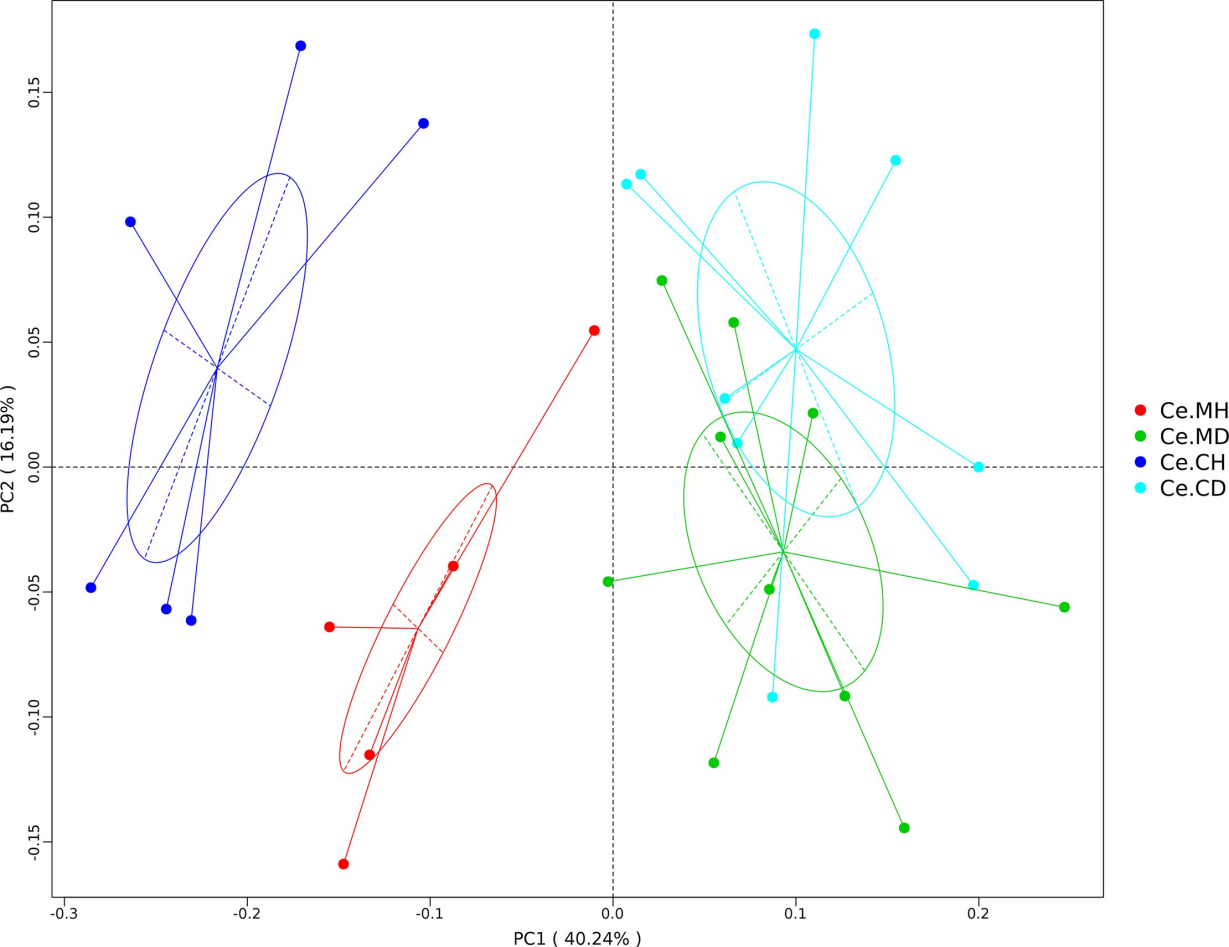
Principal coordinate analysis (PCoA) of the weighted UniFrac distances for each group. The percent variation explained by each principal coordinate is indicated on the X and Y axes.

**Fig 4:**
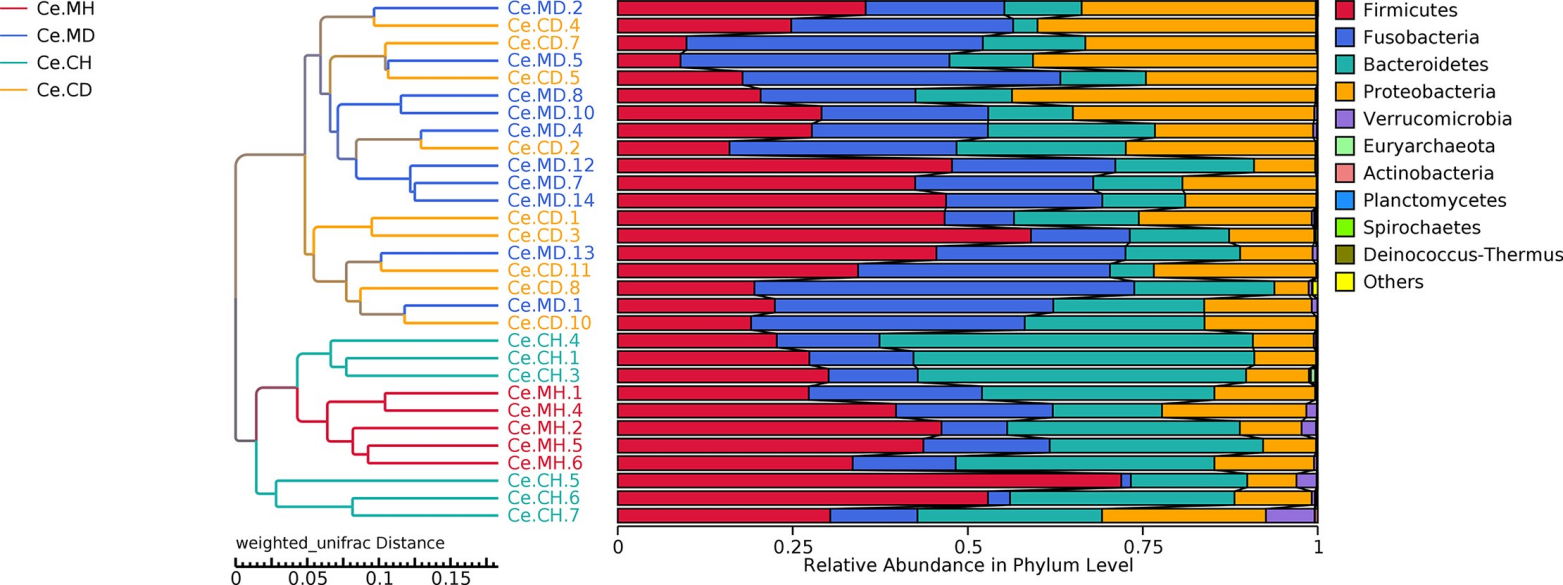
Unweighted pair-group method with arithmetic mean (UPGMA) phylogenetic tree constructed based on weighted UniFrac distance. The left panel shows the phylogenic tree, and the right panel shows the relative abundance of each sample at the phylum level.

To characterize the levels and patterns of diversity within individuals, the alpha index of the cecal microbial structure of the four groups was compared. The Shannon and Simpson indices were higher in the healthy individuals compared with those infected with PEDV (p < 0.05) (S3 Fig). Beta diversity of each group was calculated through PCoA based on weighted UniFrac distances. The PCoA scatterplot revealed clear clustering of gut bacterial communities in PEDV infection (Fig 3). All the samples were divided into the following three major clusters: the samples of the healthy groups were divided into two clusters and the samples of the infected group formed the third cluster. The distribution of samples from the healthy and infected groups obviously had a certain distance. The samples from the Ce.MD and Ce.CD groups had an intersection, indicating that the two groups have a certain similarity with respect to microbial composition. To determine the degree of similarity among the samples, the clustering tree of unweighted pair-group method with arithmetic mean (UPGMA) based on weighted UniFrac distances was constructed (Fig 4). All samples were divided into two major clusters, and the healthy group samples could be distinguished from the infected group samples using the clustering tree.

According to the MetaStat analysis, the proportion of Bacteroidetes members was significantly different between the healthy and infected groups in the cecal mucosa (S4 Fig). In addition, the difference in the proportion of Deferribacteres members between the healthy and infected groups was significant, but the relative abundance of this phylum was low in each group. According to the heatmap, there were certain differences between the groups with respect to the cluster areas of the top 30 genera at the genus level (S5 Fig). Differences in the microbial composition of each group might explain the differences in the principal coordinates.

**Fig 5:**
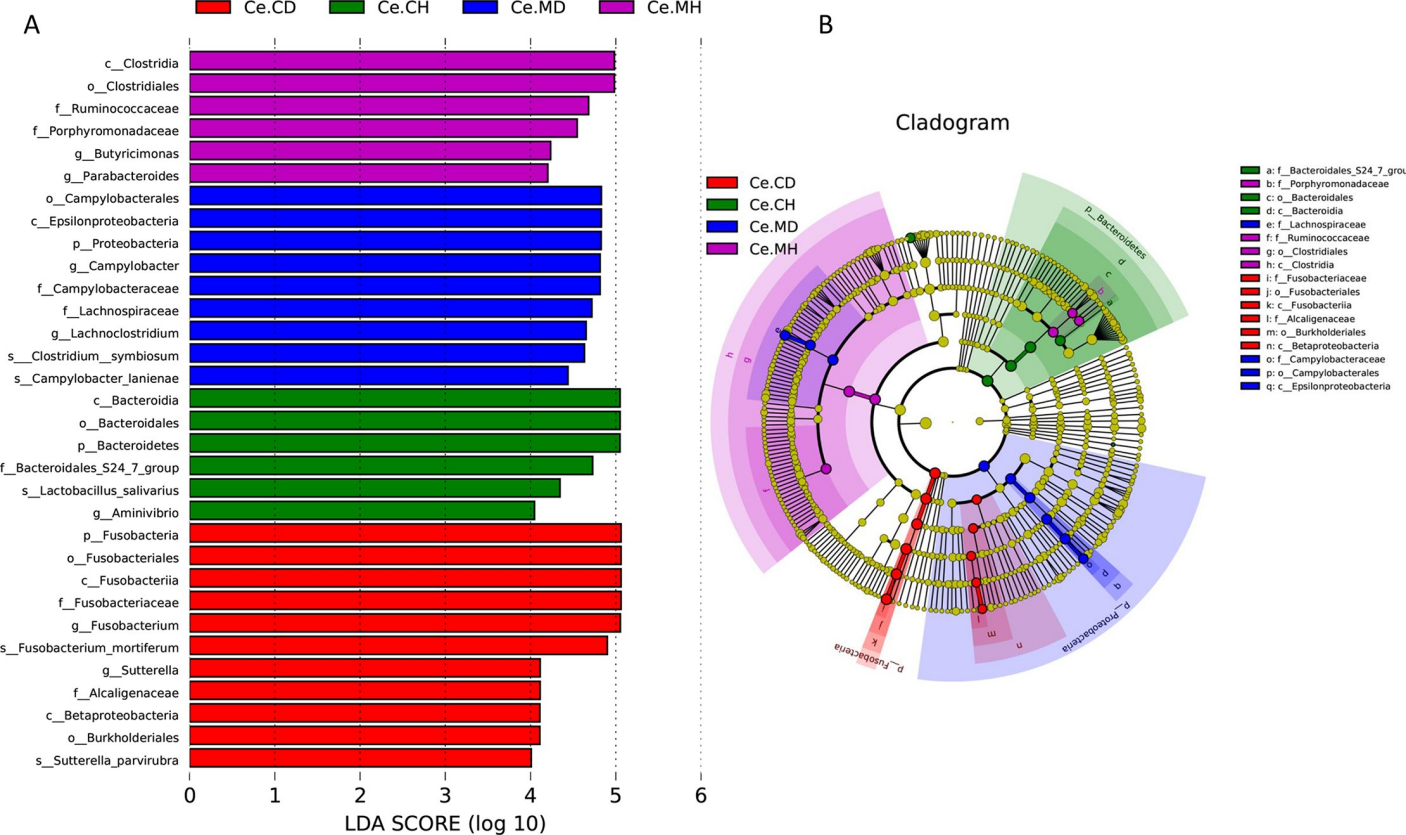
Differentially abundant microbes and taxons identified by LEfSe and the cladogram for each group. LEfSe identified taxons for Ce.CH, Ce.CD, Ce.MH and Ce.MD. (Left) Histogram of the LDA scores computed for features differentially abundant of cecal microbiota among healthy and diarrheal piglets. (Right) Taxonomic cladogram representation of statistically and biologically consistent differences of cecal microbiota among healthy and diarrheal piglets. Only taxa meeting an LDA significant threshold >4 are shown.

Using the LEfSe analysis, we evaluated the statistical difference of cecal microbial abundance from the taxonomic phylum to species (Fig 5A), and plotted a cladogram representing the structure of the host-microbiota axis. This cladogram indicated significant shifts in each of the four groups (Fig 5B). Among the four groups, 32 phylotypes from phylum to species were discovered as high-dimensional biomarkers. These microbes mainly belonged to four different phyla, namely Firmicutes, Bacteroidetes, Proteobacteria, and Fusobacteria. The genus *Butyricimonas* and *Parabacteroides* belonging to the phylum Bacteroidetes were significantly higher in abundance in Ce.MH group. The abundance of phyla Firmicutes and Proteobacteria in Ce.MD was substantially higher, due to an increase in the abundance of *Lachnoclostridium* and *Campylobacter*. The Ce.CD group had distinct cecal microbial compositions mostly belonging to phyla Fusobacteria, and was represented by *Fusobacterium* at the genus level.

### Microbial functional prediction

**Fig 6:**
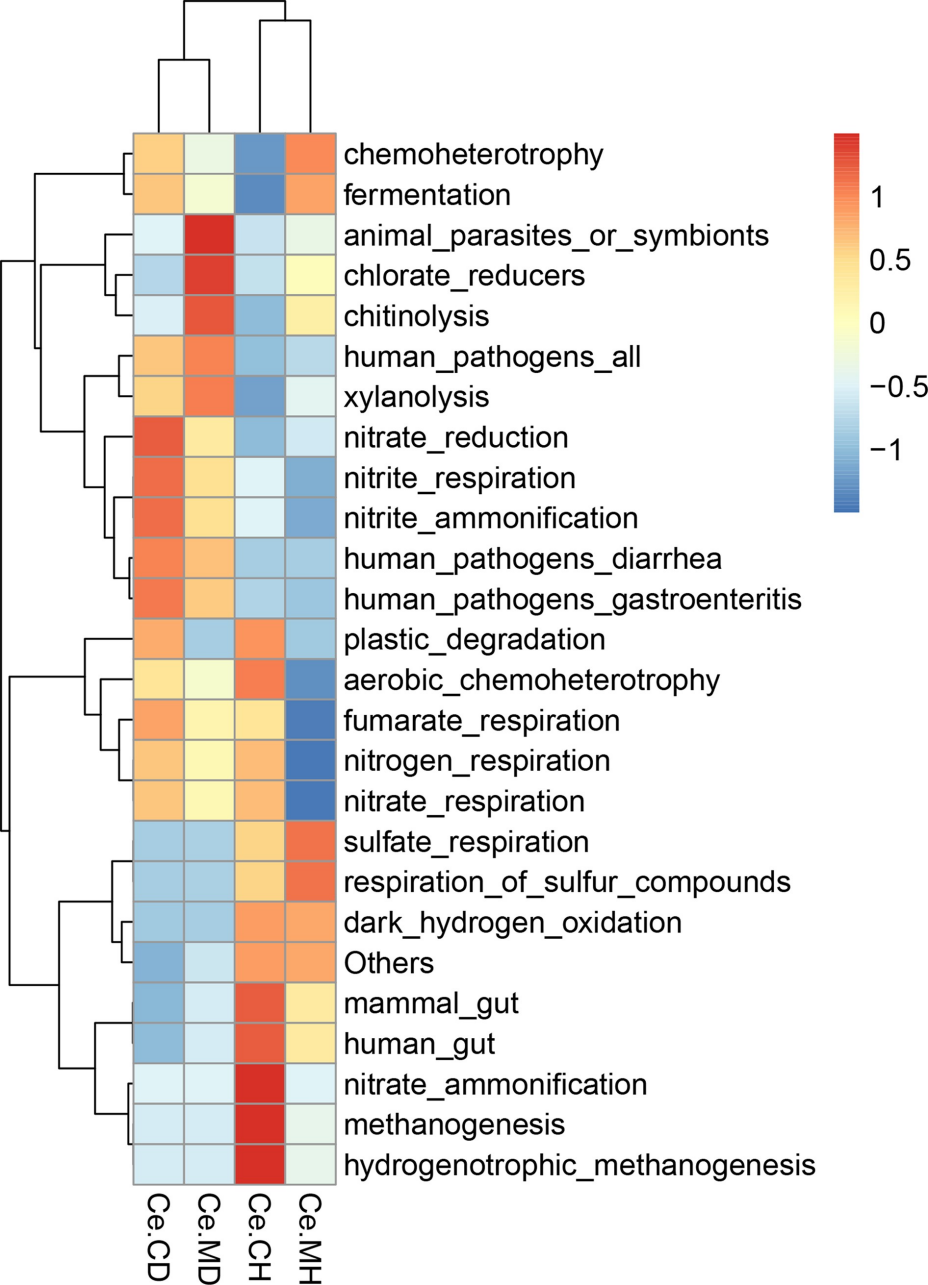
Heatmap of the functional predictions of the cecal microbiota of suckling piglets based on the bacteria in each group.

Microbial functions were predicted using FAPROTAX based on the relative abundance of microbes. Overall, 66 functional groups were identified in the cecal microbiota. “Chemoheterotrophy” and “Fermentation” were the top two functional annotations in the four groups, followed by “animal parasites or symbionts” and “mammal gut”. After functional prediction of each group, the cluster analysis was carried out using heatmap. We found that the mucosa and content microbial functional clusters were more similar in the infected groups and in the two healthy groups. Furthermore, the regions of relative abundance of each group were different (Fig 6).

## Discussion

Several microorganisms, including *Rotavirus*, *Coronavirus*, *Escherichia coli*, *Clostridium perfringens*, *Clostridium difficile*, *Cryptosporidium* spp., *Giardia* spp., *Cystoisospora suis*, and *Strongyloides ransomi*, have been linked to enteritis and diarrhea in suckling pigs. Suckling piglets are the most sensitive to PED at the age of 3–7 days [3], and the first week of birth is also a stage of gradual establishment of intestinal microorganisms [34]. The gut microbiota is relatively simple and susceptible to pathogenic bacteria [35].

In the present study, we found that healthy and PEDV-infected status had significant effects on the cecal microbiota in piglets. Our results showed that the relative proportion of Proteobacteria and Fusobacteria in the cecum microorganism of suckling piglets infected with PEDV was increased. Previous studies have also confirmed the increased presence of Fusobacteria in the feces of suckling pigs infected with PEDV [19, 20, 22]. As the main obligate anaerobic bacteria, *Fusobacteria* has been found to be involved in various clinical anaerobic infections [36, 37]. *Fusobacterium* also plays a key role in promoting colorectal cancer, and *Fusobacterium nucleatum* can cause intestinal inflammation [38, 39]. Therefore, the increased proportion of *Fusobacterium* in piglets with PEDV might cause inflammation of the cecum.

Although many studies have been conducted on pig fecal microorganisms, the direct analysis of the microbial diversity in the intestinal tract, especially the mucosa, has been rarely discussed. In our study, the cecal mucosal and content microorganisms were both selected as the research objects, and the microbial structure between healthy and PEDV-infected individuals was compared. We found that the microbial structure of samples between the Ce.CH and Ce.MH groups was obviously different. On one hand, it might be due to the insufficient sample size. On the other hand, it might be due to the instability of microbial community structure in the intestinal contents, which is largely affected by food and gastrointestinal movement, leading to fundamental changes in the intestinal contents. However, the mucosal microbes were relatively stable. In the infected groups (Ce.MD and Ce.CD), the distribution of microorganisms in the cecal mucosa and contents exhibited a certain intersection. The microbial community structure in these two groups exhibited considerable similarity. Our results showed a butyrate-producing bacterium *Butyricum* was significantly higher in abundance in Ce.MH group than others. Additionally, a reduction in *Butyricimonas* has been noted in numerous autoimmune and inflammatory diseases including inflammatory bowel disease (IBD), rheumatoid arthritis, and type 1 diabetes. Another study has also found that *Butyricimonas* was shared by all healthy piglets, but were not identified in various age groups of diarrheal piglets infected by PEDV [40]. Although the small intestine is the main site of PEDV infection, the large intestines are the main metabolic and absorption sites of microbial fermentation, with considerably more microbial abundance than the small intestines. Therefore, studying the cecum and colon is necessary to obtain useful information for overall gut responses to PEDV infection. Unlike humans, the digestive function of cecum in pigs is highly developed. Consequently, pigs can even digest crude fiber. Cecum, colon, and rectum of growing and fattening pigs are the main sites of microbial fermentation. It is generally believed that short chain fatty acids produced by microorganisms fermenting carbohydrates in cecum and colon are beneficial to host health [41].

Significantly decreased alpha diversity (Shannon and Simpson indices) in the cecal bacterial population of PEDV-infected piglets compared to the healthy ones. High diversity is considered to be a marker of mature intestinal microbiota, which is less sensitive to environmental factors and less susceptible to interference [42]. Our results suggested that the proportion of Bacteroides was decreased in the cecal microbiota of piglet infected by PEDV and that of Proteobacteria was increased. Previous studies have shown that Bacteroides and Proteobacteria play an important role in carbohydrate fermentation, polysaccharide catabolism, and amino acid and protein utilization [18, 43, 44]. The results of the LEfSe analysis showed that significant enrichment of microbes in each group was different and the cladogram results suggested that the microorganisms, which can be used as specific biomarker for each group, were also different. These microbes mainly belonged to phyla, Firmicutes, Bacteroidetes, Proteobacteria, and Fusobacteria. *Clostridium*, *Butyricimonas*, *Bacteroidetes*, and *Lactobacillus salivarius* were enriched in the healthy groups, whereas members of Proteobacteria, Campylobacter, Lachnospiraceae, and Fusobacteria were enriched in PEDV-infected groups. Some biomarkers identified by LEfSe analysis between PEDV infected and healthy groups were associated with intestinal diseases and piglet health. Therefore, the changes in the relative abundances of these bacteria and the decrease in Bacteroides proportion might partly explain the low relative abundance of microflora related to energy metabolism, secondary metabolite synthesis, and amino acid metabolism in the intestine of piglets infected with PEDV.

Healthy and infected individuals were clustered into two categories by microbial functional prediction. Porcine epidemic diarrhea virus causes intestinal malabsorption and vomiting, leading to fatal dehydration. Timely treatment of diarrhea and vomiting can effectively reduce mortality caused by PEDV [1]. Microorganisms have been used to prevent diarrhea as they play an important role in the absorption and metabolism of substances [44]. Porcine epidemic diarrhea virus changed microbial community composition in the intestine and feces of suckling piglets, including some metabolism associated microbes [20, 22, 45]. Therefore, regulating the intestinal microbiota might be a promising method to prevent or treat PEDV. However, further studies are required. Metagenomic *de novo* sequencing of the cecal mucosa and microbial genomic contents are needed to be conducted in the future to characterize the intestinal microorganisms of healthy and PEDV-infected piglets in more detail and obtain more accurate information.

## Conclusions

The results showed the abundance of bacteria associated with diseases, including diarrhea, was increased, with the abundance of beneficial bacteria decreasing. The abundance of Fusobacterium and Proteobacteria was obviously increased, and that of Bacteroidetes decreased in the PEDV-infected group. Microbial community in the cecal mucosa and contents of healthy piglets differed and PEDV infection caused similarities in the cecal mucosa and its associated microbes. The current results enhance the understanding of gut microbiota associated with PEDV.

## Supporting information

S1 Fig Detection of PEDV in healthy groups and diarrhea groups (mucosa) by qPCR. (TIF)

S2 Fig Detection of PEDV in healthy groups and diarrhea groups (content) by qPCR. (TIF)

S3 Fig Boxplot of Shannon and Simpson indices between groups. Differences between boxes were tested by Wilcoxon test (**p < 0.01, *** p < 0.0001).
(TIF)

S4 Fig Boxplot of relative abundance of Bacteroidetes in each group by metastat analysis (*p < 0.05). (TIF)

S5 Fig Heatmap analyses of abundant of top 30 genera in each group. The heatmap plot depicts the relative percentage of each bacterial genus (variables clustering on the vertical-axis) within each group (horizon-axis clustering). The color of the spots in the right panel represents the relative values (lg) of the genera in each group.
(TIF)
