# Supplementary figures and images for: Changes in cecal microbiota community of suckling piglets infected with porcine epidemic diarrhea virus
Source: PLoS One. 2019 Jul 16;14(7):e0219868. doi: 10.1371/journal.pone.0219868 (PMC6634403; doi:10.1371/journal.pone.0219868)

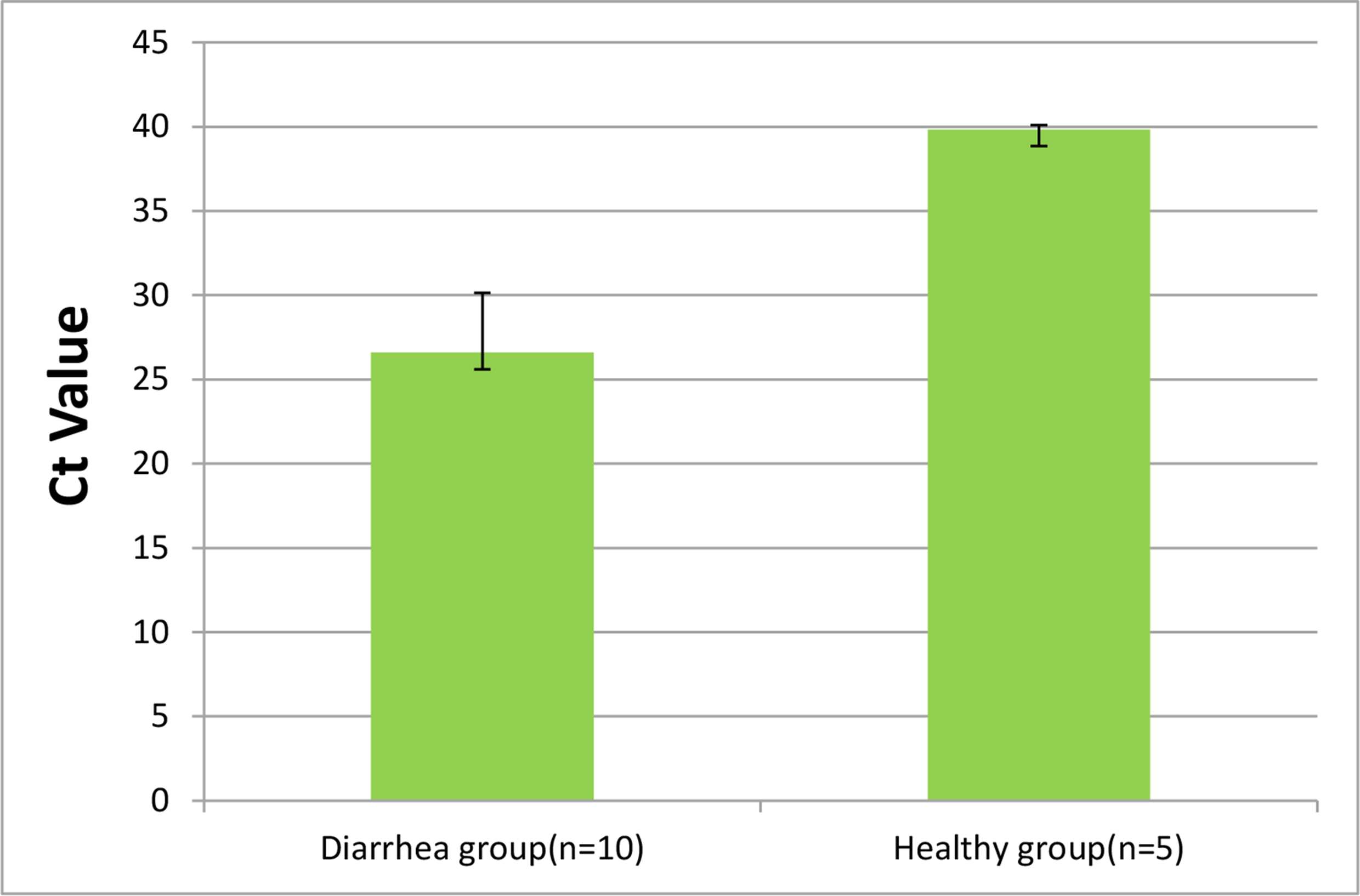

Supplement: S1 Fig — (TIF) [file pone.0219868.s001.tif]

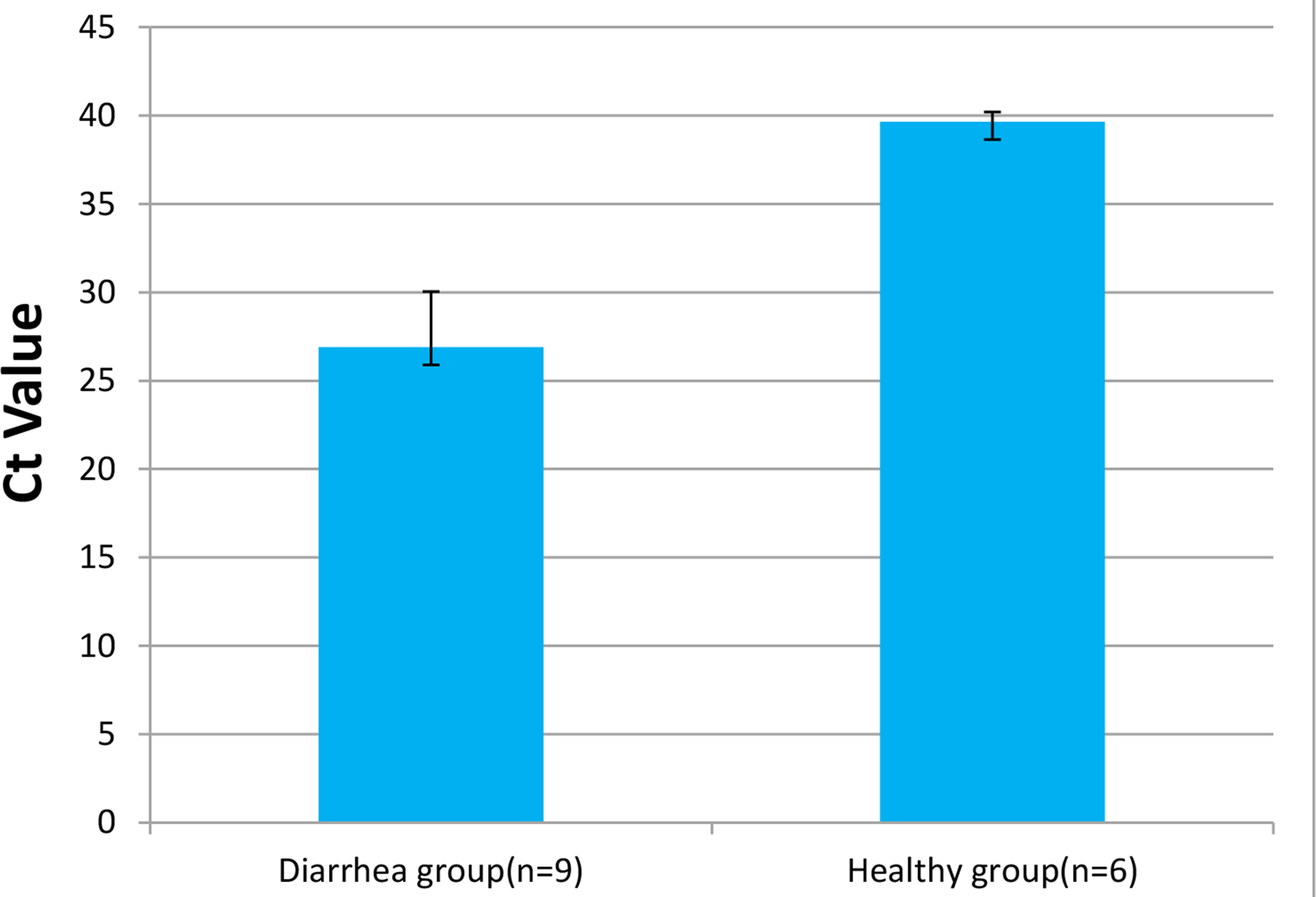

Supplement: S2 Fig — (TIF) [file pone.0219868.s002.tif]

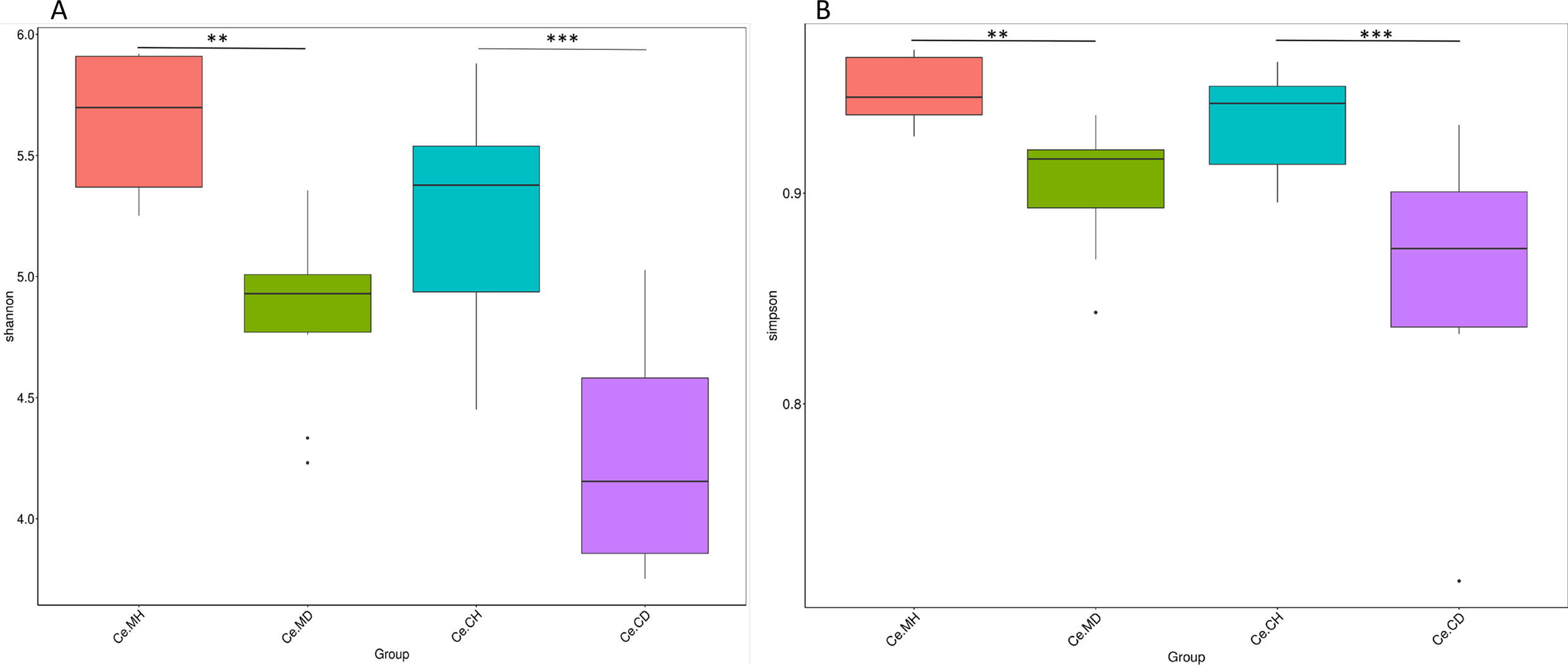

Supplement: S3 Fig — Differences between boxes were tested by Wilcoxon test (**p < 0.01, *** p < 0.0001). (TIF) [file pone.0219868.s003.tif]

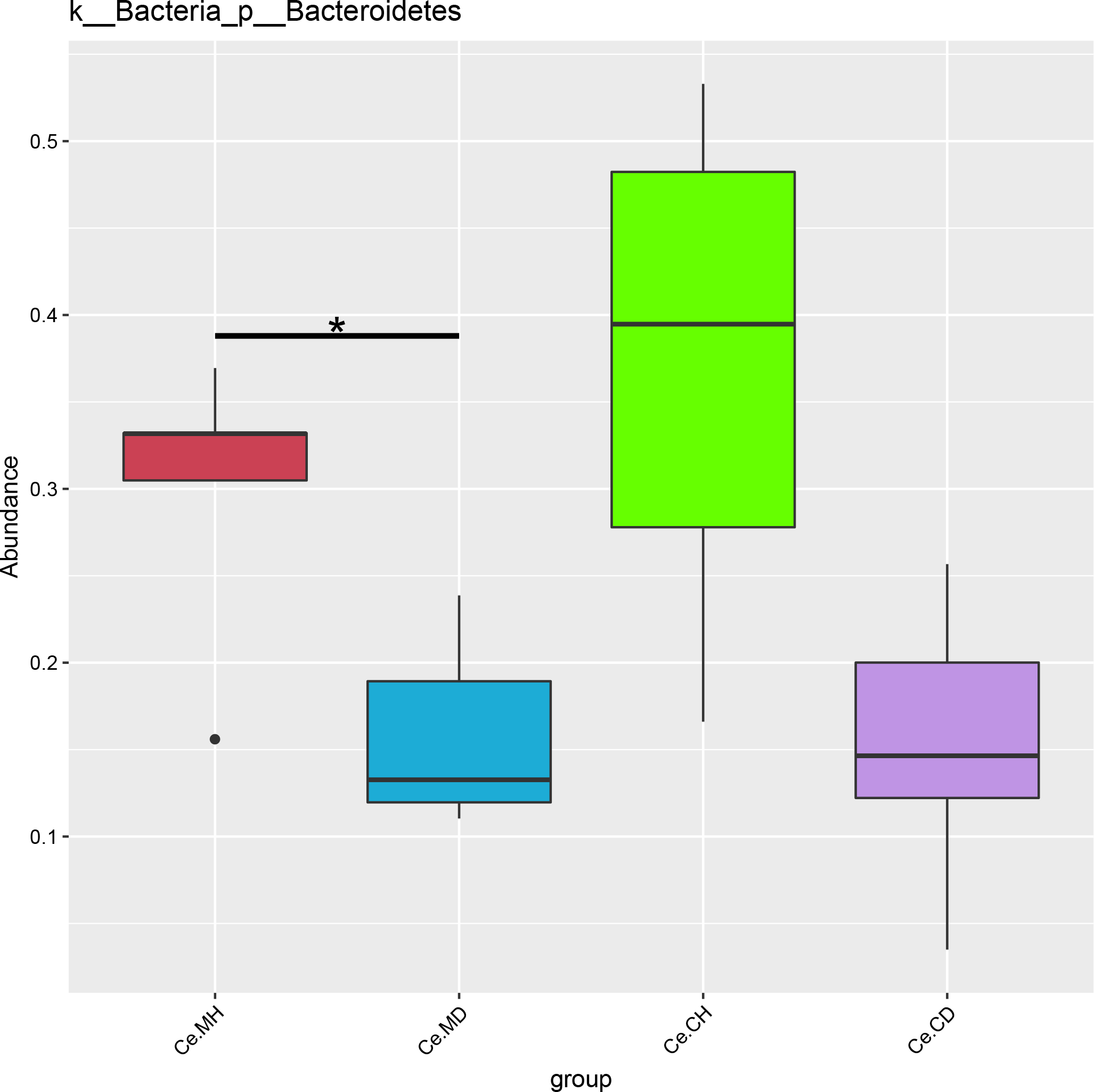

Supplement: S4 Fig — (TIF) [file pone.0219868.s004.tif]

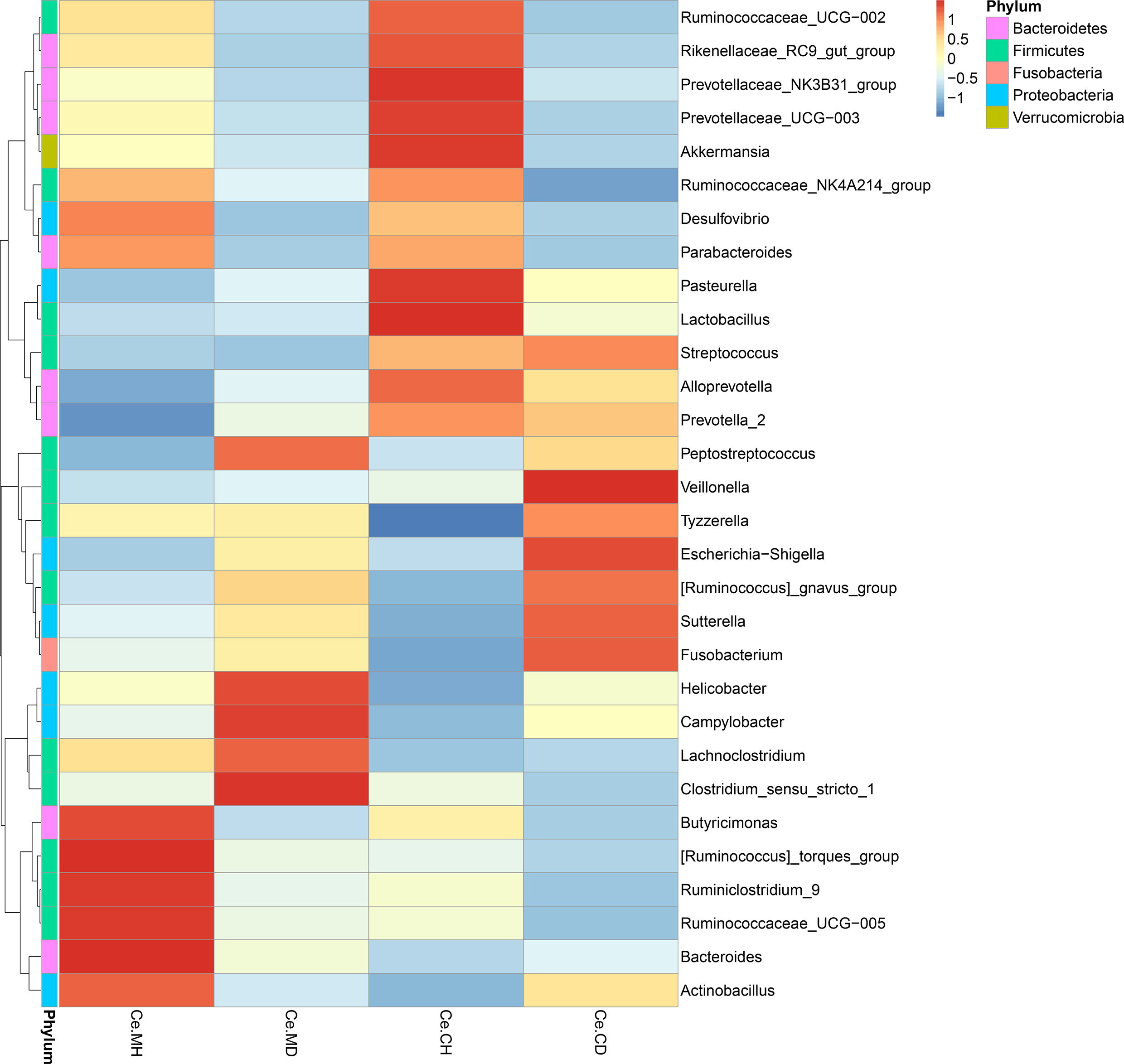

Supplement: S5 Fig — The heatmap plot depicts the relative percentage of each bacterial genus (variables clustering on the vertical-axis) within each group (horizon-axis clustering). The color of the spots in the right panel represents the relative values (lg) of the genera in each group. (TIF) [file pone.0219868.s005.tif]
